# A Textbook of Histology

**Published:** 1903-07

**Authors:** 


A TEXTBOOK OF HISTOLOGY. By DR. LADISLAUS SZYMONOWICZ, Professor of Histology and Embryology in the University of Lemberg. Translated by JOHN B. MACCALLUM, M. D., Johns Hopkins Medical School, Baltimore. Octavo, 437 pages, 277 illustrations, including 57 full-page plates in colors and monochrome. Lea Brothers & Co., Philadelphia and New York. 1902. (Cloth, $4.75 net.)

The practical importance of good works on histology, which after all is the basis of medicine and surgery, is paramount and every new work, even though it adds but very little to our knowledge of the subject, deserves attention. Many of the best works on the subject during the past few years have emanated from German sources and their translation into English has been most welcome by American readers. One of the latest works to receive such honor is Szymonowicz's histology. A work in every way entitled to the careful study and consideration.

The author divides the work as usual into two parts: Part I. treating of the microscopic anatomy of cells and tissues, and Part II. to the microscopic anatomy of the organs.

The description of the cell is carefully considered, the process of amitosis, and that of mitosis receiving considerable attention. The chapters following on the tissues—connective, nerve, muscle, bone, blood and lymph--are well written and adopted to the needs of the student of medicine.

In Part II., the different organs of the body are minutely described, and their development traced through the various stages. The illustrations accompanying the chapters on bone, muscle, skin are handsomely executed and deserve great praise. In fact, the publishers have been very liberal in the use of color illustrations and almost every chapter in Part II. has received some recognition along this line.

The book on the whole is unusually well adapted for class room purposes and for clearness of style; originality in presenting facts it stands unrivalled.

